# Detailed analysis of X chromosome inactivation in a 49,XXXXX pentasomy

**DOI:** 10.1186/1755-8166-2-20

**Published:** 2009-10-07

**Authors:** Lucia M Moraes, Leila CA Cardoso, Vera LS Moura, Miguel AM Moreira, Albert N Menezes, Juan C Llerena, Héctor N Seuánez

**Affiliations:** 1 Medical Genetics Department, Instituto Fernandes Figueira, Fiocruz, Rio de Janeiro, Brazil; 2 Genetics Division, Instituto Nacional de Câncer, Rio de Janeiro, Brazil; 3 Department of Genetics, Universidade Federal do Rio de Janeiro, Rio de Janeiro, Brazil

## Abstract

**Background:**

Pentasomy X (49,XXXXX) has been associated with a severe clinical condition, presumably resulting from failure or disruption of X chromosome inactivation. Here we report that some human X chromosomes from a patient with 49,XXXXX pentasomy were functionally active following isolation in inter-specific (human-rodent) cell hybrids. A comparison with cytogenetic and molecular findings provided evidence that more than one active X chromosome was likely to be present in the cells of this patient, accounting for her abnormal phenotype.

**Results:**

5-bromodeoxyuridine (BrdU)-pulsed cultures showed different patterns among late replicating X chromosomes suggesting that their replication was asynchronic and likely to result in irregular inactivation. Genotyping of the proband and her mother identified four maternal and one paternal X chromosomes in the proband. It also identified the paternal X chromosome haplotype (P), indicating that origin of this X pentasomy resulted from two maternal, meiotic non-disjunctions. Analysis of the *HUMANDREC *region of the androgen receptor (*AR*) gene in the patient's mother showed a skewed inactivation pattern, while a similar analysis in the proband showed an active paternal X chromosome and preferentially inactivated X chromosomes carrying the 173 *AR *allele. Analyses of 33 cell hybrid cell lines selected in medium containing hypoxanthine, aminopterin and thymidine (HAT) allowed for the identification of three maternal X haplotypes (M1, M2 and MR) and showed that X chromosomes with the M1, M2 and P haplotypes were functionally active. In 27 cell hybrids in which more than one X haplotype were detected, analysis of X inactivation patterns provided evidence of preferential inactivation.

**Conclusion:**

Our findings indicated that 12% of X chromosomes with the M1 haplotype, 43.5% of X chromosomes with the M2 haplotype, and 100% of the paternal X chromosome (with the P haplotype) were likely to be functionally active in the proband's cells, a finding indicating that disruption of X inactivation was associated to her severe phenotype.

## Background

Pentasomy X (49,XXXXX) is a rare chromosome abnormality, first described in a 2-year-old girl [1], with less than 30 cases reported in the literature [2-30] and only one in a patient of 15 years of age [25]. The characteristic phenotype associated with a 49,XXXXX karyotype is more severe than in X trisomies and tetrasomies [25,31], including severe mental retardation with delayed speech development, short stature, coarse facial features, osseous and articular abnormalities, congenital heart defects and skeletal and limb abnormalities. The actual incidence of pentasomy X is unknown but may be comparable to 49,XXXXY, occurring in 1/85,000 males [31].

In normal, 46,XX females, X chromosome inactivation is usually random [32], consequently to which every X chromosome may be inactivated in a given cell during early development; once inactivated, this state is stably maintained and transmitted to all clonal descendants [33,34]. Lyon's hypothesis [32] postulated that in patients with X chromosome polysomies, X chromosome inactivation was expected to be random and only one X chromosome would remain functionally active. However, in patients with a 49,XXXXY chromosome constitution, the late replicating X chromosomes showed different patterns of replication, suggesting that X inactivation was likely to be less efficient than in 46,XX females, and accounting for the presence of more than one active X chromosome in some cells [35,36]. Furthermore, analysis of histone H4 acetylation in a group of patients with 49,XXXXX, 49,XXXXY, 48,XXXY and 47,XXX karyotypes showed alterations in deacetylation of histone H4 once the inactive state was established [37]. These alterations might have affected the outcome in determining the number and the choice of which X chromosomes were deacetylated, probably due to the presence of more than one X chromosome undergoing inactivation. These results suggested that supernumerary X chromosomes might be associated to abnormal phenotypes due to excess of X active regions or to increased asynchronism of X chromosome replication, mainly in patients with four or five X chromosomes.

**Figure 1:**
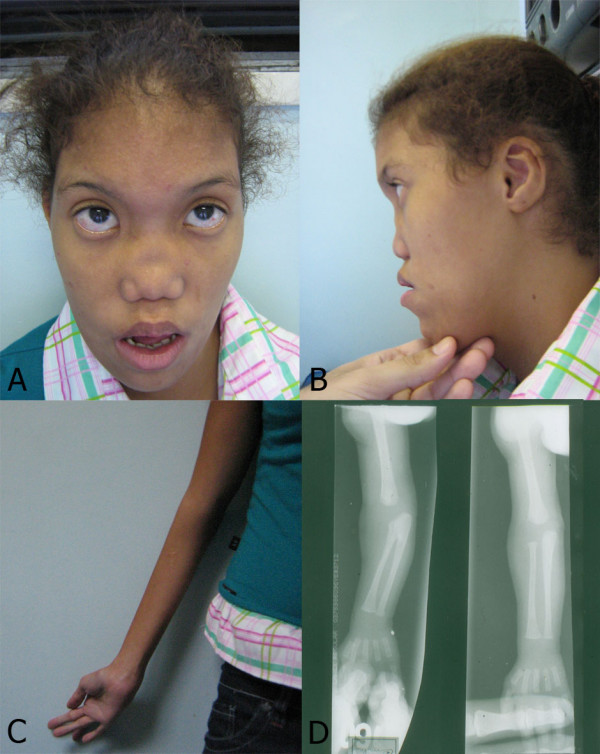
**Frontal (A) and side (B) view of patient's face**. Note flat profile, ocular hypertelorism, upslanting palpebral fissures, epicanthic folds, flat nasal bridge, low-set ears, retrognathism and micrognathia. (C) Hand arachnodactily with difficulty on supination of right arm. (D) X ray of patient's arms showing radioulnar synostosis in right arm.

In this paper, we analyzed the origin of an X chromosome pentasomy in a patient (Figure 1) with a 49,XXXXX karyotype and the inactivation status of her X chromosomes. This was carried out by analysing chromosome replication in Budr-pulsed cultures, the methylation status of the *HUMANDREC *region in the patient, her mother, and in X chromosomes present in cell hybrids previously selected in HAT (hypoxanthine-aminopterin-thymidine) medium.

## Results

### Cytogenetic analysis, X chromosome replication and parental origin of X chromosomes

**Figure 2:**
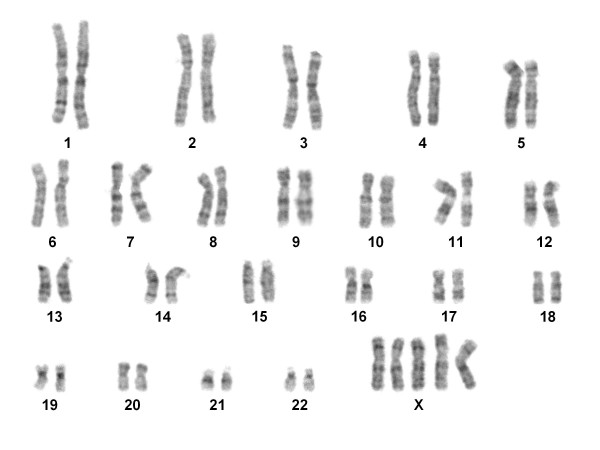
**G-band karyotype of the patient showing 49,XXXXX**.

**Figure 3:**
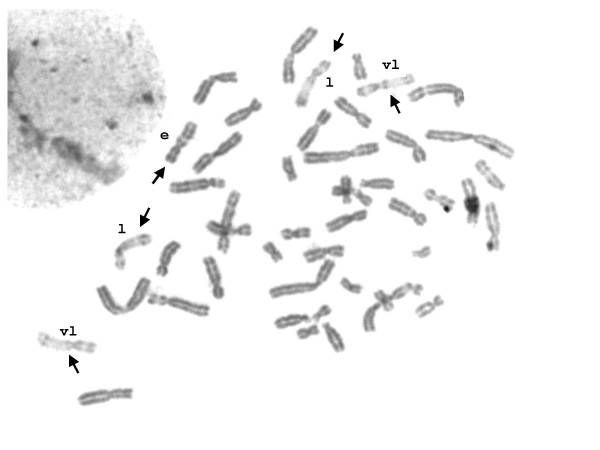
**Metaphase of BrdU-pulsed culture**. Arrows point to X chromosomes showing different replication patterns: e = early; l = late; vl = very late.

The proband showed a 49,XXXXX karyotype in all cells without evidence of mosaicism (Figure 2). 5-bromodeoxyuridine (BrdU)-pulsed cultures showed clear patterns of asynchronic replication of the proband's X chromosomes (Figure 3); in all cells one early replicating X chromosome was identified, together with four late replicating X chromosomes showing different replication patterns. Replication patterns were classified as "early replicating" (e), "late replicating" (l) and "very late replicating" (vl) according to Sarto [35]. The observed proportion of cells showing different number of X replication patterns accounted for 31% with 1e/2l/2vl, 23% with 1e/3l/1vl, 17% with 1e/1l/3vl, and 29% with 1e/4vl.

Molecular analysis of five X-linked polymorphic loci in the proband and her mother (Table 1) allowed for the unequivocal identification of the parental origin of the X chromosomes in the proband who carried four maternal X chromosomes and one paternal X chromosome; this last one with a 173-108-199-206-141 (P) haplotype.

**Table 1:** Molecular analysis of five X-linked polymorphic loci

	**MARKERS AND LOCATION**				
	
	**AFM276**	**DXS1068**	***AR***	**AFM150**	**AFM199**
	
	**(Xp21)**	**(Xp11.23)**	**(Xq11.2-q12)**	**(Xq25)**	**(Xq28)**
PROBAND (49,XXXXX)	163/185/**173**	104/112/**108**	173/179/**199**	202/204/**206**	135/139/**141**

MOTHER	163/185	104/112	173/179	202/204	135/139

Alelles are indicated by number of base pairs of amplified fragments. Paternal alleles of proband are shown in bold case.

### Preferential inactivation of X chromosomes

**Figure 4:**
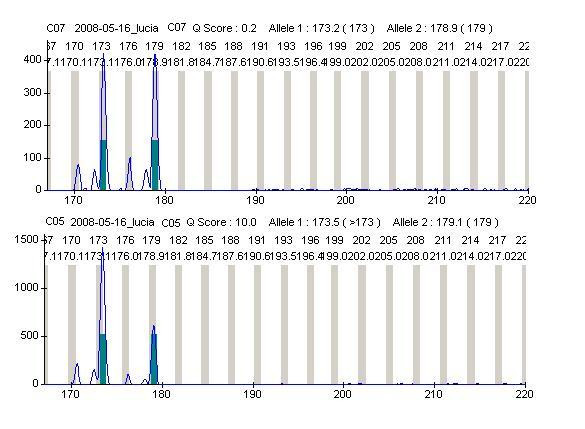
***HUMANDREC *amplification of patient's mother DNA showing alleles 173 and 179 (top)**. Amplification following digestion with methylation-sensitive endonucleases *HhaI *and *HpaII *(bottom) indicates preferential inactivation of the mother's X chromosome carrying the 173 allele.

**Figure 5:**
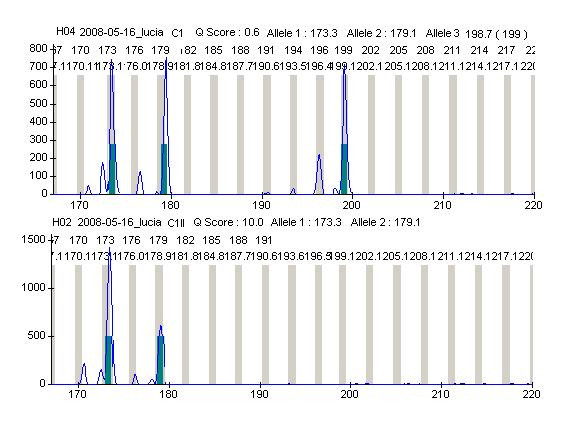
***HUMANDREC *amplification of patient's DNA showing alleles 173, 179 and 199 (top)**. Amplification following digestion with methylation-sensitive endonucleases *HhaI *and *HpaII *(bottom) indicates preferential inactivation of X chromosomes carrying the 173 allele and lack of amplification of the 199 paternal allele.

The methylation status of X chromosomes, assayed in the *HUMANDREC *region of the human androgen receptor gene (*AR*), showed a skewed pattern of inactivation in the proband's mother because one *AR *allelic fragment of 173 bp was preferentially amplified with respect to the other of 179 bp (Figure 4). Similar analysis in the proband showed that two maternal *AR *alleles were amplified (173 and 179); the 173 allele showing a preferential amplification with respect to allele 179, and lack of amplification of the paternal 199 allele (Figure 5). These results indicated (i) a preferential inactivation of maternal X chromosomes containing the 173 allele in the proband; (ii) the possibility that her maternal chromosomes with the 179 allele might be incompletely inactivated, and (iii) an active paternal X chromosome.

### Inactivation of X chromosomes present in somatic cell hybrids

The segregation of the proband's X chromosomes in a panel of 51 HPRT1^+ ^cell hybrid lines was informative for analysing the functional status of each individual X chromosome. Cell hybrids cloned in the same Petri dish were included only when they showed different molecular patterns; this was considered evidence that clones were different from one another. Conversely, when cell hybrids cloned in the same Petri dish showed an identical molecular pattern only one of them was included because we could not prove that they were different from one another. Table 2 lists the molecular patterns of 33 cell hybrid lines. Human-specific amplification patterns were not observed with rodent DNA with any primer herein tested under similar PCR conditions.

**Table 2:** Molecular patterns of 33 cell hybrid lines and X chromosome haplotypes

**Hybrid cell**	**Human X chromosome marker**					**Haplotype(s)**
		
	**AFM276**	**DXS1068**	***AR***	**AFM150**	**AFM199**	
1PXKA1	185/163	112/104	173/179	202/204	135/139	M1-M2

1PXK A3	185/163/**173**	112/104/**108**	173/179/**199**	202/204/**206**	135/139/**141**	M1-M2-P

1PXKB1	185/163	112/104	173/179	202/204	135/139	M1-M2

1PXKB2	185/**173**	112/**108**	173/**199**	202/**206**	135/**141**	M1-P

1PXKB4	185/163/**173**	112/104/**108**	173/179/**199**	202/204/**206**	135/139/**141**	M1-M2-P

1PXKB5	185	112	173	202	135	M1

1PXKC1	185/163	112/104	173/179	202/204	135/139	M1-M2

1PXKC2	185/163/**173**	112/104/**108**	173/179/**199**	202/204/**206**	135/139/**141**	M1-M2-P

1PXKE2	185/163	112/104	173/179	202/204	135/139	M1-M2

1PXKE3	**173**	**108**	**199**	**206**	**141**	P

1PXKE4	185/163/**173**	112/104/**108**	173/179/**199**	202/204/**206**	135/139/**141**	M1-M2-P

1PXKF1	185/163	112/104	173/179	202/204	135/139	M1-M2

1PXKF4	185/**173**	112/**108**	173/**199**	202/**206**	135/**141**	M1-P

1PXKG4	163	104	179	204	139	M2

1PXKH2	163/**173**	104/**108**	179/**199**	204/**206**	139/**141**	M2-P

1PXKH4	185	112	173	202	135	M1

1PXKI1	185/163/**173**	112/104/**108**	173/179/**199**	202/204/**206**	135/139/**141**	M1-M2-P

1PXKI3	185/**173**	112/**108**	173/**199**	202/**206**	135/**141**	M1-P

1PXKJ6	163	104	179	204	139	M2

1PXKL1	185/**173**	112/**108**	173/**199**	202/**206**	135/**141**	M1-P

1PXKL3	185/163/**173**	112/104/**108**	173/179/**199**	202/204/**206**	135/139/**141**	M1-M2-P

1PXKM1	163/**173**	104/**108**	179/**199**	204/**206**	139/**141**	M2-P

1PXKM3	185/163/**173**	112/104/**108**	173/179/**199**	202/204/**206**	135/139/**141**	M1-M2-P

1PXKN1	185/163	112/104	173/179	202/204	135/139	M1-M2

1PXKN2	185/**173**	112/**108**	173/**199**	202/**206**	135/**141**	M1-P

1PXKO1	185/163/**173**	112/104/**108**	173/179/**199**	202/204/**206**	135/139/**141**	M1-M2-P

1PXKP3	185/163	112/104	173/179	202/204	135/139	M1-M2

1PXKQ2	185/163/**173**	112/104/**108**	173/179/**199**	202/204/**206**	135/139/**141**	M1-M2-P

1PXKQ3*	185/**173**	112/**108**	179/**199**	204/**206**	139/**141**	MR-P

1PXKR1	163	104	179	204	139	M2

1PXKR4	185/163	112/104	173/179	202/204	135/139	M1-M2

1PXKS1	185/163	112/104	173/179	202/204	202/204	M1-M2

1PXKS3	185/163/**173**	112/104/**108**	173/179/**199**	202/204/**206**	135/139/**141**	M1-M2-P

Paternal alleles are shown in bold case; * = cell hybrid with recombinant X haplotype (MR)

**Table 3:** X chromosome inactivation in cell hybrids with more than one X haplotype

**Cell hybrid**	**Haplotypes**	**Number of X chromosomes**	***AR *alelles**	**Amplified alelle(s)**
1PXKA1	M1-M2	2 (92%), 1(8%)	173/179	173 = 179

1PXKA3	M1-M2-P	3 (75%), 2 (18%), 1 (7%)	173/179/**199**	173 > 179

1PXKB1	M1-M2		173/179	173

1PXKB2	M1-P	2 (88%), 1 (12%)	173/**199**	173

1PXKB4	M1-M2-P		173/179/**199**	173 > 179

1PXKC1	M1-M2		173/179	173

1PXKC2	M1-M2-P		173/179/**199**	173 > 179

1PXKE2	M1-M2		173/179	173

1PXKE4	M1-M2-P		173/179/**199**	173 > 179

1PXKF1	M1-M2		173/179	173

1PXKF4	M1-P	2 (85%), 1 (15%)	173/**199**	173

1PXKH2	M2-P	2 (86%), 1 (14%)	179/**199**	179

1PXKI1	M1-M2-P		173/179/**199**	173 > 179

1PXKI3	M1-P		173/**199**	173

1PXKL1	M1-P		173/**199**	173

1PXKL3	M1-M2-P		173/179/**199**	173 > 179

1PXKM1	M2-P	2 (89%), 1 (11%)	179/**199**	179

1PXKM3	M1-M2-P		173/179/**199**	173 > 179

1PXKN1	M1-M2		173/179	173

1PXKN2	M1-P		173/**199**	173

1PXKO1	M1-M2-P		173/179/**199**	173 > 179

1PXKP3	M1-M2		173/179	179

1PXKQ2	M1-M2-P		173/179/**199**	173 > 179

1PXKQ3*	MR-P	2 (84%), 1 (16%)	179/**199**	179

1PXKR4	M1-M2		173/179	173

1PXKS1	M1-M2		173/179	173

1PXKS3	M1-M2-P	3 (78%), 2 (14%), 1 (8%)	173/179/**199**	173 > 179

Paternal alleles are shown in bold case. Number of X chromosomes was estimated by FISH

Analysis of cell hybrids allowed for the identification of three maternal haplotypes: M1, with 185-112-173-202-135 (in cell hybrids 1PXKB2, 1PXKB5, 1PXKF4, 1PXKH4, 1PXKI3, 1PXKL1, 1PXKN2); M2, with 163-104-179-204-139 (in cell hybrids 1PXKG4, 1PXKH2, 1PXKJ6, 1PXKM1 and 1PXKR1), and MR, with 185-112-179-204-139 (in cell hybrid 1PXKQ3). MR was a recombinant maternal haplotype, derived from a crossover between DXS1068 and *AR*. The finding of cell hybrid lines with single M1, M2 or P haplotypes, indicated that the *HPRT1 *locus of donor origin was functionally active in different X chromosomes of the proband.

To discriminate whether survival in HAT medium was associated to presence of one (or more) active X chromosome(s) in cell hybrids showing any combination of two or three haplotypes, DNA from cell hybrids was first digested with methylation-sensitive enzymes and subsequently amplified with AR-C primers (see Table 3).

These results showed that in nine cell hybrids with M1-M2 haplotypes, the 173 allele was exclusively amplified in seven cell lines. In one cell hybrid line (1PXKA1), both maternal *AR *alleles (173 and 179) were amplified, showing similar peaks and suggesting random inactivation, while in another cell hybrid line (1PXKP3), the 179 allele was exclusively amplified. In all five cell hybrids with the M1-P haplotypes, the 173 maternal allele was exclusively amplified, as was the 179 maternal allele in the two M2-P cell hybrid lines. Similarly, the 179 maternal allele was exclusively amplified in 1PXKQ3, a cell hybrid with a recombinant maternal haplotype (MR) and a paternal (P) haplotype. In ten other cell hybrid lines with the M1-M2-P haplotypes, the observed pattern of amplification was similar to the one observed in the proband's lymphocytes, showing a large peak corresponding to allele 173, a smaller peak corresponding to allele 179, and lack of amplification of the 199 paternal allele.

### Number of X chromosomes in selected cell hybrids

**Figure 6:**
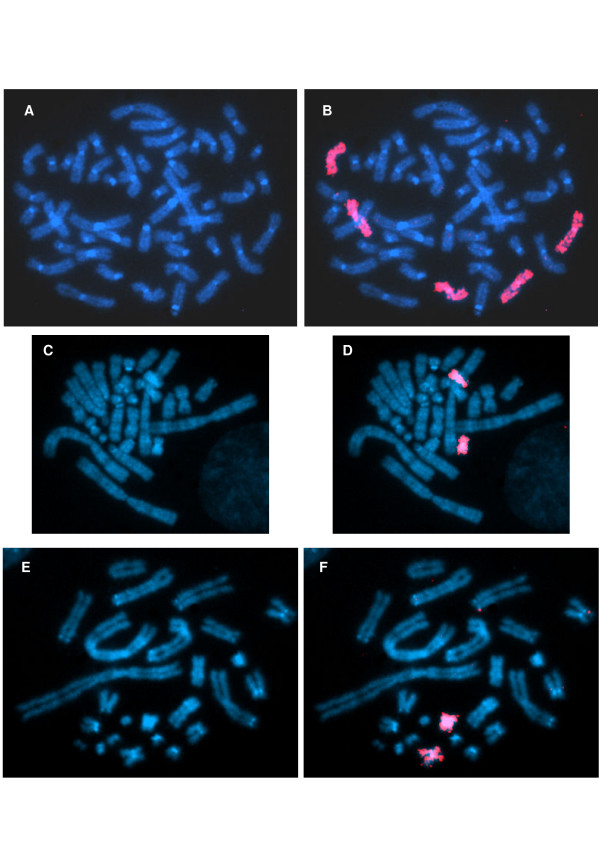
**A-B: Chromosome painting of a metaphase of the patient showing presence of five X chromosomes**. C-D: Chromosome painting of a metaphase of cell hybrid 1PXKH2 showing two human X chromosomes. E-F: Chromosome painting of a metaphase of cell hybrid 1PXKA1 showing two human X chromosomes.

A comparison with FISH data showed that the number of human X chromosomes retained in cell hybrids and the number of X haplotypes were very frequently coincident in a sample of eight cell hybrid lines in which all possible haplotype combinations were observed Table 3; Figure 6). This correspondence was also found in cell hybrid 1PXKJ6, in which a single haplotype and a single human X chromosome were observed.

### Analysis of a region of the *XIST *promoter

Sequence analysis of the minimal region of the *XIST *promoter did not show C(- 43)A or C(- 43)G in the proband and her mother, ruling out that alterations at this site might be responsible for skewed X inactivation. Moreover, mutational screening of the proband's X chromosomes in 10 cell hybrids confirmed that these mutations were not present in her chromosomes. This was especially evident in three of these cell hybrids (1PXKB5, 1PXKE3 and 1PXKG4), each which with a single X haplotype (M1, P and M2 respectively).

## Discussion

Here we report the second case of a 49,XXXXX karyotype in a proband of 15 years of age [25] and the first one analyzed in detail for its X inactivation pattern. Analysis of BrdU-pulsed cultures suggested that the functionally status of the four allocyclic X chromosomes varied within and between cells, probably resulting from disruption of the kinetics of late DNA replication and accounting for the a higher number of active X chromosome regions in the 49,XXXXX patient with respect to the single inactivated X chromosome in a normal 46,XX female.

The molecular identification of four maternal X chromosomes in the proband indicated that this maternal X tetrasomy originated by two consecutive non disjunctions during oogenesis, in agreement with previous findings on the maternal origin of X polysomies [20,21,29,38]. This was in agreement with the subsequent identification, in the cell hybrid panel, of three maternal haplotypes, two presumably parental (M1 and M2) and one recombinant (MR). Altogether, the proband carried four different maternal X chromosomes; the fourth one corresponding to the MR counterpart (with the 163-104-173-202-135 haplotype) that was not found in the cell hybrid panel.

A comparison of X inactivation patterns in the proband showed that her paternal X chromosome was always active and that her maternal X chromosomes carrying the 173 allele (with M1 and the 163-104-173-202-135 haplotypes) were preferentially inactivated with respect to X chromosomes with 179 allele (with M2 and MR haplotypes). Interestingly, the smaller peak of the amplified 179 allele indicated that some X chromosomes containing this allele had not been inactivated.

Analysis of cell hybrids confirmed these findings in 10 cell hybrids with M1-M2-P haplotypes, while in seven cell hybrids with M1-M2 haplotypes, 173 was the only amplified allele. However, it also showed that not all X chromosomes with the M1 haplotype were inactivated because (i) M1 was shown to be the only haplotype in two cell hybrids (1PXKB5 and 1PXKH4); (ii) in one M1-M2 cell hybrid (1PXKP3), only the 179 allele was amplified, and (iii) an apparent random inactivation was observed in another M1-M2 cell hybrid (1PXKA1), indicated by an equal amplification of 173 and 179 alleles (Table 3). Furthermore, M2 was the only haplotype found in three cell hybrids (1PXKG4, 1PXKJ6 and 1PXKR1; Table 2), indicating that their X chromosomes were functionally active. Moreover, in ten cell hybrids with the M1-M2-P haplotypes, inactivation patterns suggested that X chromosomes with the M2 haplotype were not completely inactivated.

When counting the number of haplotypes associated to X chromosome activity, demonstrated by positive selection in HAT medium, there was a 100% correspondence between the P haplotype and X chromosome activity in all 19 cell hybrids where this haplotype was identified. Conversely, the M1 haplotype, present in 25 cell hybrids, was associated to X chromosome activity in only three cell hybrids (12%), while the M2 haplotype, present in 23 cell hybrids, was associated to activity in 10 cell hybrids (43.5%). Finally, the MR haplotype was not found to be associated with X chromosome activity in our cell hybrid panel.

FISH analysis showed a reasonable correspondence between number of different haplotypes and number of X chromosomes in nine cell hybrid lines although this coincidence did not prove that these X chromosomes were originally present in the same human cells from which each cell hybrid derived because cell fusion does not necessarily take place between single human cells and single rodent cells.

## Conclusion

Our studies showed that, under the hypothesis of random X inactivation, the theoretically expected five equal classes of cells with one active X chromosome and four inactive X chromosomes were unlikely to be present in the proband because the paternal X chromosome was always active, accounting for a 5-fold increase with respect to its expected proportion (20%). Moreover, 12% of X chromosomes with the M1 haplotype were shown to be active, while the maternal X chromosome with M2 haplotype was active in 43.5% of cell hybrids, in more than twice the expected proportion assuming random inactivation. It is likely that the presence of five X chromosomes might drastically affect the process of inactivation that was found to require transient localization of X inactivation centres in cell nuclei [39] and correct pairing between X chromosomes. Furthermore, we did not find alterations at the - 43 *XIST *minimal promoter [40,41] that might explain the skewed inactivation pattern observed in the proband or her mother which, however, did not rule out that mutations might have occurred at other promoter regions.

Finally, the presence of five X chromosomes in the proband must have represented a serious misbalance in early stages of development before the time X inactivation occurred. Moreover, this unusual high number of X chromosomes must have subsequently impaired the process of X dosage compensation, consequently to which more than one X chromosome remained functionally active, resulting in a functional X polysomy. These factors must account for her severe physical phenotype and mental retardation, probably aggravated by a parental imprinting effect [42] resulting from active X chromosomes of the same parental origin.

### Proband and Methods

C.S.S is a 15 year old female whose mother was 17 years of age at the time she was born. At birth, she weighted 1,200 g and her height was 39 cm. She was referred for clinical investigation for multiple anomalies (congenital heart defect, cleft soft palate, and facial dysmorphies) at 4 months of age. A recent physical examination showed a mentally retarded young adult, with poor language development, marfanoid habitus and disproportionate upper/lower body segments (Figure 1); her profile was very flat; with ocular hypertelorism, upslanting palpebral fissures, epicanthic folds, flat nasal bridge, low-set ears, posterior cleft palate, retrognathism, and micrognathia. A low posterior hairline, left scoliosis, bilateral fifth finger clinodactily, hand and feet arachnodactily, and difficulty on supination of the right arm corresponding to a radioulnar synostosis were present. A congenital heart defect (patent ductus arteriosus and ventricular septal defect) was surgically repaired at the first year of life. A low count of dermal ridges was also present.

Cytogenetic analyses were carried out at 4 months and at 15 years of age using standard lymphocyte cultures and BrdU-pulsed cultures [43]. Chromosome preparations were identified by conventional GTG banding [44] at the 500 band level in 100 cells. Chromosome preparations of BrdU-pulsed cultures were stained [45], and X replication patterns identified in 100 cells and classified according to Sarto's criteria [35].

### Cell Fusion protocols

Hprt - cell lines of rodent origin were used as recipient cell lines in fusion experiments with human lymphocytes of the proband isolated with Ficoll^® ^(Sigma, St Louis). A cell hybrid panel was prepared with AKO1/15 cells derived from *Akodon cursor *[46]. Following a seven day growth in DMEM/6MP selective medium (Dulbecco's Minimal Essential Medium supplemented with 10% FCS and 6-mercaptopurine 16.7 μg/ml), 3 × 10^6 ^recipient cells were fused with 1.2 × 10^7 ^human lymphocytes [47]. Cell hybrids were selected in DMEM/HAT medium [DMEM with 10% FCS, hypoxanthine (100 μM), aminopterin (0.4 μM), thymidine (16 μM)].

Cell death was monitored periodically; after a 2-3 weeks period, visible colonies were cloned inside Penicillinders, transferred to individual dishes and expanded to culture flasks. A maximum of three colonies was cloned from each Petri dish. Each dish was identified by a letter and each colony by a number. Controls were carried out for demonstrating adverse selection of recipient cells; aliquots of the same AKO1/15 cells used in fusion experiments were grown in DMEM/HAT medium.

### DNA isolation and PCR amplifications of X linked polymorphic markers

DNA was extracted from blood samples of the proband, her mother, the recipient cell line AKO1/15, and cell hybrid lines. DNA isolation was carried out by standard procedures [48].

PCR amplifications of four human, X-linked microsatellite loci were carried out with DNA samples of the proband, her mother, 51 cell hybrid lines, and the rodent cell line. PCR reactions contained 100 ng of DNA, reaction buffer [16 mM (NH_4_)_2_SO_4_, 67 mM Tris-HCl (pH = 8.8), 0.01% Tween-20, 2.5 mM MgCl_2_], 0.1 mM each dNTP, 5 pM of each primer and 0.6 U *Taq *polymerase (Pharmacia) in a final volume of 15 μl. All microsatellite forward-primers were 5'-labelled with FAM fluorochrome (Perkin-Elmer). Cycling conditions included an initial denaturation at 94°C for 5 min and pairing at 65°C for 5 min, followed by 30 cycles at 94°C for 45 sec; 60°C with primer pairs DXS1068.PCR1 and AFM150xf10 (or 65°C with primer pairs AFM276xf5 and AFM199wc7) for 45 sec; 72°C for 45 sec, and a final extension period of 20 min at 72°C.

Amplified products were run in a MEGABACE 1000 automatic DNA sequencer with 10× LPA buffer and MegaBACE™ Long Read Matrix with the GT Dye set 2 ET-ROX, FAM (Applied Biosystems) and analyzed with Genetic Profiler^® ^v.2.0 (Amersham, 2002).

### Analysis of X chromosome inactivation patterns

X-inactivation patterns were analyzed in the proband, her mother and in 35 cell hybrid lines previously shown to contain two or more X-chromosome haplotypes. The methylation status of the X chromosome was assayed in a region of the human androgen receptor (*AR*) gene located in Xq11-q12. This *AR *region corresponded to nucleotides 451-661 of the *HUMANDREC *sequence [49] (GenBank M20132), containing restriction sites for the methylation-sensitive endonucleases *HhaI *and *HpaII *and a highly polymorphic VNTR. Polymorphisms were initially detected in undigested genomic DNA samples with a 5' FAM-labelled, forward AR-C primer, under specific PCR conditions [50]. X chromosome methylation was assayed by separate digestions of genomic DNA with excess of *HhaI *and *HpaII *in final volumes of 22.5 μl at 37°C for 18 hours. Digestion products were subsequently used in PCR reactions, under identical conditions with those used for detecting VNTR polymorphisms. All PCR products were analyzed with Genetic Profiler^®^, version 2.0.

### Fluorescence *in situ *hybridization (FISH)

The X pentasomy was also demonstrated by FISH with an X whole chromosome probe. The number of X chromosomes present in cell hybrid lines 1PXKA1, 1PXKA3, 1PXKB2, 1PXKF4, 1PXKH2, 1PXKJ6, 1PXKM1, 1PXKQ3 and 1PXKS3 was estimated in 100 cells per cell line. In all cell hybrid lines (except for 1PXKJ6), more than one X haplotype was identified, which were present in different combinations (M1-M2, M1-P, M2-P, MR-P and M1-M2-P).

We used a human specific, X chromosome probe (WCPX) provided by Roscoe Stanyon (Frederick Cancer Research Facility, NCI, NIH), which was amplified and labelled by random priming with the 6MW primer 5'-CCG ACT CGA GNN NNN NAT GTG G-3' (N = any base). Reactions were carried out with 150 ng of probe DNA, 20 pmol of primer, 5 μl of 10× reaction buffer (245 mM TAPs, 500 mM KCl, 20 mM MgCl_2_, 2 mM dithiothreitol; pH = 9.3); 0.5 mM dCTP, dATP, dGTP, 0.25 mM dTTP; 5 μl of W-1 detergent (stock solution = 100 μl of W-1 in 10 ml of distilled water), 2 nM FITC-12-dUTP (Roche), and 3 U of *Taq Platinum*^® ^(Invitrogen), in final volumes of 50 μL. Cycling conditions consisted of an initial denaturation at 94°C for 3 min followed by 35 cycles at 94°C for 1 min, a 62°C for 1 min, 72°C for 1 min, and a final extension period at 72°C for 9 min.

Slides were denatured in 70% formamide/2× SSC (pH = 7.0) at 73°C for 5 min and dehydrated in 70%, 85% and 100% ethanol and allowed to dry at room temperature. Probe preparation, incubation with slides and further processing were carried out according to Vysis^® ^standard protocols for hybridization with whole chromosome probes. Preparations were analyzed with epifluorescence microscopes (Olympus DX-60 and DX-50). Images were captured with QUIPS - PathVysion (Vysis^®^) and Applied Imaging.

### Analysis of the minimal region of the *XIST *promoter

Analysis of a 380 pb minimal region of the *XIST *promoter was carried for characterising position - 43 with respect to the possibility of finding C(- 43)A or C(- 43)G in the proband, her mother and in ten cell cell hybrid lines derived from the proband (1PXKA1, 1PXKB1, 1PXKB2, 1PXKB5, 1PXKC1, 1PXKE3, 1PXKG4, 1PXKJ6, 1PXKP3, 1PXKQ2). DNA was amplified with a forward primer 5'-TGAGAACTGGAAAACCCATTG-3' and a reverse primer 5'-ATACGCCATAAAGGGTGTTGG-3'. Reactions were carried out with 200 ng of DNA, 25 pmol of each primer, 0.2 mM of each dNTP and 0.2 U of *Taq Platinum^® ^*(Invitrogen), with 1× enzyme buffer and 1.5 mM MgCl_2_, in a final volumes of 50 μL. Cycling conditions included an initial denaturation step at 94°C for 5 min, followed by 35 cycles at 94°C for 45 sec, 55°C for 45 sec, and 72°C for 45 sec, with a final extension period at 72°C for 15 min in a GeneAmp PCR System 2400 (Applied Biosystems). Amplified products were purified with GFX PCR DNA and Gel Band Purification Kit (GE Healthcare) and eluted in 50 μL of water. Approximately 100 ng of the purified fraction was labelled with 5 pmol of either primer according to the conditions outlined by the DYEnamic ET Dye Terminator Cycle Sequencing Kit for MegaBace DNA Analysis Systems (GE Healthcare). Sequencing was carried out in a MEGABACE 1000 automatic DNA sequencer. Sequences were aligned manually with Chromas version 1.45 [51] and MEGA 4.0 [52].

## Abbreviations

HAT: **H**ypoxanthine, **A**minopterin, **T**hymidine; FISH: **F**lourecence ***i****n ****s****itu ***h**ybridization; BrdU: bromo-deoxy-uridine; GTG banding: G-banding following trypsin digestion and Giemsa staining; DMEM: Dulbecco's Minimal Essential Medium; FCS: foetal calf serum; 6MP: 6-mercaptopurine; VNTR: **V**ariable **N**umber of **T**andem **R**epeats

## Competing interests

The authors declare that there are no competing interests (financial or non financial), and that the interpretation of data has no been influenced by anyone.

## Authors' contributions

LMM carried out the cytogenetic studies and DNA sequencing and created the cell hybrid panel. LCAC carried out the assays for identifying X inactivation with blood and cell hybrid DNAs. MAMM participated in the design of experiments and interpretation of data. ANM participated in genotyping with microsatellite markers. VLSM and JCL Jr. attended the patient at the Pediatric clinic. HNS participated in the design of experiments, interpretation of data and drafted the manuscript. All authors read and approved the final version of the manuscript.

## Consent

Written informed consent was obtained from the patient's mother for publication of this case report and accompanying images. A copy of the written consent is available for review by the Editor-in-Chief of this journal.

## References

[B1] Kesaree N, Woolley PV (1963). A Phenotypic Female with 49 Chromosomes, Presumably XXXXX. a Case Report. J Pediatr.

[B2] Ricci N, Dallapiccola B, Ventimiglia B, Tiepolo L, Fraccaro M (1968). 48,XXXX-49,XXXXX mosaic: asynchronies among the late-replicating X chromosomes. Cytogenetics.

[B3] Zajaczkowska K, Korniszewski L, Wolff-Plodowska A (1970). A case of quintuple-X syndrome (49,XXXXX). J Ment Defic Res.

[B4] Sergovich F, Uilenberg C, Pozsonyi J (1971). The 49,XXXXX chromosome constitution: similarities to the 49,XXXXY condition. J Pediatr.

[B5] Yamada Y, Neriishi S (1971). Penta X (49,XXXXX) chromosome constitution: a case report. Jinrui Idengaku Zasshi.

[B6] Cooke P, Black JA, Curtis DJ (1972). Comparative clinical studies and X chromosome behaviour in a case of XXXX-XXXXX mosaicism. J Med Genet.

[B7] Larget-Piet L, Rivron J, Baillif P, Dugay J, Emerit I, Larget-Piet A, Berthelot J (1972). 49, XXXXX syndrome in a 5-year-old girl. Ann Genet.

[B8] Giovannucci-Uzielli ML, Torricelli F, Salvatori Q, Consumi I, Donzelli GP, Seminara S (1975). 49, XXXXX chromosome equipment in a girl with psychophysical underdevelopment. Minerva Pediatr.

[B9] Tumba A, Fryns JP, van OG, Berghe H van den (1977). 49,XXXXX syndrome: apropos of a further case. Union Med Can.

[B10] Kukharenko VI, Grinberg KN, Kuliev AM (1978). Mitotic cycles in human cell strains with sex chromosomes aneuploidy. Hum Genet.

[B11] Archidiacono N, Rocchi M, Valente M, Filippi G (1979). X pentasomy: a case and review. Hum Genet.

[B12] Carpenter DG, Connolly JM, Carter CH, Kanarek KS (1979). The penta X (49,XXXXX) syndrome: danger of confusing phenotype with mongolism. Am J Dis Child.

[B13] Dryer RF, Patil SR, Zellweger HU, Simpson JM, Hanson JW, Aschenbrenner C, Weinstein SL (1979). Pentasomy X with multiple dislocations. Am J Med Genet.

[B14] Monheit A, Francke U, Saunders B, Jones KL (1980). The penta-X syndrome. J Med Genet.

[B15] Schroeter C, Jahrig K, Weinke I (1980). A new case of pentasomy X. Helv Paediatr Acta.

[B16] Funderburk SJ, Valente M, Klisak I (1981). Pentasomy X: report of patient and studies of X-inactivation. Am J Med Genet.

[B17] Fragoso R, Hernandez A, Plascencia ML, Nazara Z, Martinez y Martinez R, Cantu JM (1982). 49,XXXXX syndrome. Ann Genet.

[B18] Zhang RH, Pan NH, Li XF, Wang XQ, Wu M (1982). A case of 49, XXXXX syndrome. Chin Med J.

[B19] Gomez-Valencia L, Najera-Martinez P, Morales-Hernandez A, Martinez-Diaz De Leon A (1989). Penta-X syndrome. Report of a case with 47,XXX/48,XXXX/49,XXXXX mosaicism. Bol Med Hosp Infant Mex.

[B20] Hassold T, Pettay D, May K, Robinson A (1990). Analysis of non-disjunction in sex chromosome tetrasomy and pentasomy. Hum Genet.

[B21] Deng HX, Abe K, Kondo I, Tsukahara M, Inagaki H, Hamada I, Fukushima Y, Niikawa N (1991). Parental origin and mechanism of formation of polysomy X: an XXXXX case and four XXXXY cases determined with RFLPs. Hum Genet.

[B22] Kassai R, Hamada I, Furuta H, Cho K, Abe K, Deng HX, Niikawa N (1991). Penta X syndrome: a case report with review of the literature. Am J Med Genet.

[B23] Nakano S, Sasame A, Azukizawa S, Kigoshi T, Uchida K, Takahashi H, Morimoto S (1992). Pentasomy X mosaic in two adult sisters with diabetes mellitus. Intern Med.

[B24] Martini G, Carillo G, Catizone F, Notarangelo A, Mingarelli R, Dallapiccola B (1993). On the parental origin of the X's in a prenatally diagnosed 49,XXXXX syndrome. Prenat Diagn.

[B25] Linden MG, Bender BG, Robinson A (1995). Sex chromosome tetrasomy and pentasomy. Pediatrics.

[B26] Myles TD, Burd L, Font G, McCorquodale MM, McCorquodale DJ (1995). Dandy-Walker malformation in a fetus with pentasomy X (49,XXXXX) prenatally diagnosed by fluorescence in situ hybridization technique. Fetal Diagn Ther.

[B27] Boeck A, Gfatter R, Braun F, Fritz B (1999). Pentasomy X and hyper IgE syndrome: co-existence of two distinct genetic disorders. Eur J Pediatr.

[B28] Biroli E, Ghimenti C, Ricci I, Pirola B, Liverani ME, Perona A, Galligani L, Guala A, Angeli G (2003). Sex chromosome abnormality: report of three clinical cases of X pentasomy. Pathologica.

[B29] Cho YG, Kim DS, Lee HS, Cho SC, Choi SI (2004). A case of 49,XXXXX in which the extra X chromosomes were maternal in origin. J Clin Pathol.

[B30] Cheng PJ, Chueh HY, Shaw SW, Hsu JJ, Hsieh TT, Soong YK (2008). X pentasomy in an intracytoplasmic sperm injection pregnancy detected by nuchal translucency testing. Fetal Diagn Ther.

[B31] Kleczkowska A, Fryns JP, Berghe H Van den (1988). X-chromosome polysomy in the male. The Leuven experience 1966-1987. Hum Genet.

[B32] Lyon MF (1961). Gene action in the X-chromosome of the mouse (*Mus musculus *L.). Nature.

[B33] Takagi N, Sasaki M (1975). Preferential inactivation of the paternally derived X chromosome in the extraembryonic membranes of the mouse. Nature.

[B34] Willard H (1995). Sex chromosomes and X chromosome inactivation. The Metabolic and Molecular Bases of Inheritance Disease.

[B35] Sarto GE, Otto PG, Kuhn EM, Therman E (1987). What causes the abnormal phenotype in a 49,XXXXY male?. Hum Genet.

[B36] Therman E, Denniston C, Sarto GE, Ulber M (1980). X chromosome constitution and the human female phenotype. Hum Genet.

[B37] Leal CA, Ayala-Madrigal ML, Figuera LE, Medina C (1998). Histone H4 acetylation analyses in patients with polysomy X: implications for the mechanism of X inactivation. Hum Genet.

[B38] Visootsak J, Rosner B, Dykens E, Tartaglia N, Graham JM (2007). Behavioral phenotype of sex chromosome aneuploidies: 48,XXYY, 48,XXXY, and 49,XXXXY. Am J Med Genet.

[B39] Bacher CP, Guggiari M, Brors B, Augui S, Clerc P, Avner P, Eils R, Heard E (2006). Transient colocalization of X-inactivation centres accompanies the initiation of X inactivation. Nat Cell Biol.

[B40] Plenge RM, Hendrich BD, Schwartz C, Arena JF, Naumova A, Sapienza C, Winter RM, Willard HF (1997). A promoter mutation in the *XIST *gene in two unrelated families with skewed X-chromosome inactivation. Nat Genet.

[B41] Bicocchi MP, Migeon BR, Pasino M, Lanza T, Bottini F, Boeri E, Molinari AC, Corsolini F, Morerio C, Acquila M (2005). Familial nonrandom inactivation linked to the X inactivation centre in heterozygotes manifesting haemophilia A. Eur J Hum Genet.

[B42] Iitsuka Y, Bock A, Nguyen DD, Samango-Sprouse CA, Simpson JL, Bischoff FZ (2001). Evidence of skewed X-chromosome inactivation in 47,XXY and 48,XXYY Klinefelter patients. Am J Med Genet.

[B43] Zakharov AF, Baranovskaia LI, Ibraimov AI (1974). Differential condensation of human chromosomes in mitosis under the influence of 5-bromdesoxycytidine. Tsitologiia.

[B44] Seabright M (1971). A rapid banding technique for human chromosomes. Lancet.

[B45] Perry P, Wolff S (1974). New Giemsa method for the differential staining of sister chromatids. Nature.

[B46] Bonvicino CR, Moreira MA, Arcuri RA, Seuánez HN (2001). Induction and characterization of hypoxanthine-phosphoribosyltransferase (Hprt-) deficient cell lines of *Akodon cursor *(Rodentia, Sigmodontinae). Cytogenet Cell Genet.

[B47] Rivero MB, Olicio R, Lima CR, Bonvicino CR, Moreira MA, Llerena JC, Seuánez HN (2001). Molecular analysis of HPRT1(+) somatic cell hybrids derived from a carrier of an *HPRT1 *mutation responsible for Lesch-Nyhan syndrome. Am J Med Genet.

[B48] Sambrook J, Fritsch EF, Maniatis T (1989). Molecular Cloning: A Laboratory Manual.

[B49] Lubahn DB (1988). The human androgen receptor: complementary deoxyribonucleic acid cloning, sequence analysis and gene expression in prostate. Mol Endocrinol.

[B50] Delabesse E, Aral S, Kamoun P, Varet B, Turhan AG (1995). Quantitative non-radioactive clonality analysis of human leukemic cells and progenitors using the human androgen receptor (*AR*) gene. Leukemia.

[B51] MacCarthy C (1998). Chromas, version 1.45.

[B52] Tamura K, Dudley J, Nei M, Kumar S (2007). MEGA4: Molecular Evolutionary Genetics Analysis (MEGA) software version 4.0. Mol Biol Evol.

